# Supplementary material for: Supporting parents in the Global South: implementation of a faith-based parent program in 12 countries
Source: Glob Ment Health (Camb). 2025 Mar 5;12:e37. doi: 10.1017/gmh.2025.25 (PMC11949733; doi:10.1017/gmh.2025.25)
Supplement: Rojas-Flores et al. supplementary material 1 — Rojas-Flores et al. supplementary material [file S2054425125000251sup001.docx]

***Supplementary Materials:***  *Table S6. Percentage of Recorded Caregiver Workshop Attendance*

| Percentage Attendance | 0% | 1-49% | 50-74% | 75-99% | 100% | Total |
| --- | --- | --- | --- | --- | --- | --- |
| Philippines | 16 | 2 | 36 | 0 | 150 | 204 |
| Asia Total | 16 | 2 | 36 | 0 | 150 | 204 |
| DRC | 12 | 1 | 5 | 0 | 160 | 178 |
| Ethiopia | 82 | 0 | 0 | 0 | 160 | 242 |
| Ghana | 2 | 0 | 0 | 1 | 198 | 201 |
| Kenya | *No attendance data available* | | | | | 0 |
| Mozambique | 34 | 0 | 0 | 0 | 164 | 198 |
| Rwanda | 42 | 0 | 1 | 0 | 117 | 160 |
| Zimbabwe | 107 | 3 | 19 | 0 | 121 | 250 |
| Africa Total | 279 | 4 | 25 | 1 | 920 | 1229 |
| El Salvador | 94 | 4 | 9 | 8 | 50 | 165 |
| Guatemala | 52 | 24 | 20 | 31 | 73 | 200 |
| Honduras | 0 | 0 | 0 | 25 | 175 | 200 |
| Nicaragua | 54 | 14 | 48 | 0 | 85 | 201 |
| Central America Total | 200 | 42 | 77 | 64 | 383 | 766 |
| Total | 495 | 48 | 138 | 65 | 1453 | 2199 |

***Supplementary Materials****: Table S7.* *Facilitator Self-Report of Implementation Fidelity for CF Parent Workshops*

| Region | Africa | | | | | | | Central America | | | | Asia |
| --- | --- | --- | --- | --- | --- | --- | --- | --- | --- | --- | --- | --- |
| Country | Ethiopia | Ghana | Kenya | Mozambiqe | Rwanda | Zimbabwe | DRC | El Salvador | Guatemala | Honduras | Nicaragua | Philippines |
| Prepared all necessary documents | 8/8 (100%) | 9/9 (100%) | 5/5 (100%) | 14/15  (93%) | 8/8 (100%) | 7/7 (100%) | Data not available | 18/18 (100%) | 9/9  (100%) | 20/20 (100%) | 15/15 (100%) | 19/19 (100%) |
| Attendance collected | 8/8 (100%) | 9/9 (100%) | 5/5 (100%) | 14/15  (93%) | 8/8 (100%) | 7/7 (100%) | - | 18/18 (100%) | 9/9  (100%) | 20/20 (100%) | 15/15 (100%) | 19/19 (100%) |
| Followed instructions for each module | 8/8 (100%) | 9/9 (100%) | 5/5 (100%) | 15/15 (100%) | 8/8 (100%) | 7/7 (100%) | - | 18/18 (100%) | 9/9 (  100%) | 20/20 (100%) | 15/15 (100%) | 19/19 (100%) |
| Understood material | 8/8 (100%) | 9/9 (100%) | 5/5 (100%) | 15/15 (100%) | 8/8 (100%) | 7/7 (100%) | - | 18/18 (100%) | 9/9  (100%) | 20/20 (100%) | 15/15 (100%) | 19/19 (100%) |
| Modified curriculum as necessary | 8/8 (100%) | 9/9 (100%) | 5/5 (100%) | 15/15 (100%) | 8/8 (100%) | 7/7 (100%) | - | 7/18  (39%) | 6/9  (67%) | 20/20 (100%) | Data not available | 19/19 (100%) |
| Snacks provided | 8/8 (100%) | 9/9 (100%) | 5/5 (100%) | 15/15 (100%) | 8/8 (100%) | 7/7 (100%) | - | 13/18  (72%) | 6/9  (67%) | 2/20 (10%) | Data not available | 19/19 (100%) |
| Childcare provided | 8/8 (100%) | 9/9 (100%) | 5/5 (100%) | 15/15 (100%) | 8/8 (100%) | 5/7  (71%) | - | 9/18  (50%) | 3/9  (33%) | 14/20 (70%) | Data not available | 0/19  (0%) |
| Identified parent support group leaders | 8/8 (100%) | 9/9 (100%) | 5/5 (100%) | 15/15 (100%) | 8/8 (100%) | 7/7 (100%) | - | 15/18  (83%) | 8/9  (89%) | 20/20 (100%) | 12/15 (80%) | 16/19 (84%) |

*Note.* Only one fidelity checklist was submitted for DRC CF Parent Workshops. Additionally, Nicaragua did not submit information about modifications, snacks, or childcare.

***Supplementary Materials****: Table S8.* Caregiver *Change in Harsh Parenting Behaviors*

|  | *Harsh Parenting Behaviors*  *T1 to T3* | | | | | |
| --- | --- | --- | --- | --- | --- | --- |
|  | *n* | T1  *M* (SE) | T3  *M* (SE) | *M*  diff. | *p* | Cohen's *d* |
| Africa | 1188 | 2.01 (0.06) | 0.64 (0.04) | 1.37 | < .001 | 0.75 |
| DRC | 137 | 2.91 (0.19) | 1.62 (0.16) | 1.28 | < .001 | 0.62 |
| Ethiopia | 157 | 2.48 (0.20) | 0.34 (0.07) | 2.14 | < .001 | 1.13 |
| Ghana | 191 | 1.90 (0.15) | 0.99 (0.10) | 0.90 | < .001 | 0.50 |
| Kenya | 195 | 2.74 (0.19) | 0.66 (0.09) | 2.08 | < .001 | 0.97 |
| Mozambique | 157 | 0.76 (0.10) | 0.26 (0.05) | 0.50 | < .001 | 0.51 |
| Rwanda | 158 | 1.41 (0.15) | 0.08 (0.03) | 1.34 | < .001 | 0.92 |
| Zimbabwe | 193 | 1.86 (0.14) | 0.58 (0.08) | 1.28 | < .001 | 0.82 |
| Central America | 332 | 1.11 (0.08) | 0.11 (0.02) | 1.00 | < .001 | 0.94 |
| El Salvador | 12 | 1.08 (0.23) | 0.83 (0.21) | 0.25 | 0.46 | 0.33 |
| Guatemala | 122 | 0.32 (0.07) | 0.09 (0.03) | 0.23 | < .05 | 0.39 |
| Honduras | 148 | 1.78 (0.13) | 0 (0.00) | 1.78 | < .001 | 1.58 |
| Nicaragua | 50 | 1.02 (0.19) | 0.30 (0.12) | 0.72 | < .001 | 0.64 |
| Asia |  |  |  |  |  |  |
| Philippines | 148 | 2.16 (0.13) | 1.18 (0.12) | 0.97 | < .001 | 0.64 |
|  |  |  |  |  |  |  |
| All | 1668 | 1.84 (0.05) | 0.58 (0.03) | 1.26 | < .001 | 0.73 |

***Supplementary Materials****: Table S9.* *Household participation in other World Vision activities in the past year*

| Region | Africa  (n = 1277) | Central America  (n = 725) | Asia (Philippines only)  (n = 199) |
| --- | --- | --- | --- |
| Sponsorship | 52.55% | 48.97% | 73.87% |
| Economic Development (livelihood) | 32.65% | 13.79% | 59.30% |
| Education | 40.80% | 35.72% | 80.90% |
| Health | 34.22% | 25.93% | 75.88% |
| Nutrition | 24.90% | 20.00% | 74.87% |
| Disaster Risk Reduction | 22.63% | 10.62% | 65.33% |
| Child Protection | 39.23% | 19.72% | 77.89% |
| Child Participation | 37.74% | 25.24% | 83.42% |
| Peace Building | 25.84% | 15.17% | 65.33% |
| Leadership Development | 24.35% | 10.34% | 60.30% |
| Spiritual Nurture | 32.97% | 23.86% | 81.41% |
