# Supplementary material for: Supporting parents in the Global South: implementation of a faith-based parent program in 12 countries
Source: Glob Ment Health (Camb). 2025 Mar 5;12:e37. doi: 10.1017/gmh.2025.25 (PMC11949733; doi:10.1017/gmh.2025.25)
Supplement: Rojas-Flores et al. supplementary material 2 — Rojas-Flores et al. supplementary material [file S2054425125000251sup002.docx]

***Supplementary Materials***

*Figure 1. Attrition for Africa*

Baseline child/youth surveys

**1240**

Dyads enrolled

**1240**

Baseline caregiver surveys

**1277**

**Baseline**

Pre-workshop surveys

**1056**

**CF Parent Workshop**

Workshop Attendance

0% **(279)**

1-49% **(4)**

50-79% **(25)**

80-100% **(921)**

Post-workshop surveys

**1186**

*Figure 2. Attrition for Philippines*

Baseline child/youth surveys

**155**

Dyads enrolled

**155**

Baseline caregiver surveys

**199**

**Baseline**

Pre-workshop surveys

**151**

**CF Parent Workshop**

Workshop Attendance

0% **(16)**

1-49% **(2)**

50-79% **(36)**

80-100% **(150)**

Post-workshop surveys

**137**

*Figure 3. Attrition for Central America*

Baseline child/youth surveys

**640**

Dyads enrolled

**640**

Baseline caregiver surveys

**725**

**Baseline**

Pre-workshop surveys

**644**

**CF Parent Workshop**

Workshop Attendance

0% **(200)**

1-49% **(42)**

50-79% **(77)**

80-100% **(447)**

Post-workshop surveys

**445**
